# Healthcare providers on the frontlines: a qualitative investigation of the social and emotional impact of delivering health services during Sierra Leone’s Ebola epidemic

**DOI:** 10.1093/heapol/czw055

**Published:** 2016-06-08

**Authors:** Shannon A. McMahon, Lara S. Ho, Hannah Brown, Laura Miller, Rashid Ansumana, Caitlin E. Kennedy

**Affiliations:** ^1^The Institute of Public Health, Faculty of Medicine, University of Heidelberg, Heidelberg, 69120, Germany; ^2^Department of International Health, Johns Hopkins Bloomberg School of Public Health, Baltimore, MD, 21205, USA; ^3^International Rescue Committee, New York, NY, 10017, USA; ^4^Department of Anthropology, Durham University, Durham, DH1 3LE, UK; ^5^International Rescue Committee, Freetown, Sierra Leone; ^6^Department of Parasitology, Faculty of Biological Sciences, Liverpool School of Tropical Medicine, Liverpool, L35QA, UK; ^7^Department of Community Health and Clinical Studies, School of Community Health Sciences, Njala University, Bo, Sierra Leone; ^8^Mercy Hospital Research Laboratory, Bo, Sierra Leone

**Keywords:** Ebola, frontline health workers, mental health and psychosocial support, qualitative research, Sierra Leone

## Abstract

Although research on the epidemiology and ecology of Ebola has expanded since the 2014–15 outbreak in West Africa, less attention has been paid to the mental health implications and the psychosocial context of the disease for providers working in primary health facilities (rather than Ebola-specific treatment units). This study draws on 54 qualitative interviews with 35 providers working in eight peripheral health units of Sierra Leone's Bo and Kenema Districts. Data collection started near the height of the outbreak in December 2014 and lasted 1 month. Providers recounted changes in their professional, personal and social lives as they became *de facto* first responders in the outbreak. A theme articulated across interviews was Ebola’s destruction of social connectedness and sense of trust within and across health facilities, communities and families. Providers described feeling lonely, ostracized, unloved, afraid, saddened and no longer respected. They also discussed restrictions on behaviors that enhance coping including attending burials and engaging in physical touch (hugging, handshaking, sitting near, or eating with colleagues, patients and family members). Providers described infection prevention measures as necessary but divisive because screening booths and protective equipment inhibited bonding or ‘suffering with’ patients. To mitigate psychiatric morbidities and maladaptive coping mechanisms—and to prevent the spread of Ebola—researchers and program planners must consider the psychosocial context of this disease and mechanisms to enhance psychological first aid to all health providers, including those in peripheral health settings.

Key Messages

- This article examines the social and emotional experiences of front-line providers (those not working in Ebola-specific treatment facilities) as they became *de facto* first responders in Sierra Leone's Ebola outbreak.
- Frontline healthcare workers described how Ebola weakened a sense of trust within and across health facilities, providers, communities and households.
- Along with changes in their professional lives, communities and homes, providers described a profound sense of stigmatization, suffering, loneliness, isolation and sadness since the onset of Ebola.
- To mitigate psychiatric morbidities and maladaptive coping mechanisms, health systems must consider how to enhance mental health and psychosocial support for not only providers working in designated Ebola treatment and care facilities but also those working in facilities that are not specifically for Ebola management.

## Introduction

As of 10 January 2016, Sierra Leone reported 14 122 cases (including 8704 laboratory-confirmed cases) of Ebola virus disease (Ebola), the highest number of reported cases amid the ongoing West Africa outbreak (CDC 2016). During the outbreak, an unprecedented number of doctors, nurses and healthcare workers have been infected (Fischer *et al.* 2014; WHO 2014b). The WHO estimated in April 2015 that 303 health workers in Sierra Leone had been infected with Ebola, and 221 had died (WHO 2015a). In Liberia and Guinea, health worker fatalities due to Ebola were 188 and 94, respectively (WHO 2015a).

A small but growing body of literature from the current epidemic has emphasized that efforts to address Ebola outbreaks—during, immediately after and in the longer-term—must be more attentive to the mental health needs and psychosocial context of the disease (Arwady *et al.* 2014; Cooper 2015; Hughes 2015; Lee-Kwan *et al.* 2014). This sentiment is echoed in recommendations from earlier Ebola outbreaks in Uganda (Kinsman 2012) and Democratic Republic of Congo (De Roo *et al.* 1998) and from outbreaks of yellow fever and cholera in the 18th and 19th centuries (Honigsbaum 2014).

Existing literature at the nexus of mental health and Ebola focuses on patients and, to a lesser extent, their families. Those studies highlight how individuals enduring Ebola often face stigmatization, depression, economic adversity and—in the case of survivors and their families—survivor's guilt and non-acceptance upon reintegration into society (Arwady *et al.* 2014; De Roo *et al.* 1998; Lee-Kwan *et al.* 2014). Stigma has been particularly highlighted throughout history in relation to illnesses that are life-threatening, difficult to treat and whose source and transmission mechanism are (at least initially) uncertain; HIV/AIDS is a historically recent example (Wainberg *et al.* 2014). The current Ebola outbreak has highlighted stigmatization at local, regional and global levels. In Sierra Leone specifically, an August 2014 survey conducted by UNICEF, Catholic Relief Services and Focus 1000 found that in a random sample of 1413 population-based respondents, 96% held discriminatory attitudes towards those who had or were suspected of having Ebola, 76% of respondents said they would not welcome someone back in their community if they had recovered from Ebola and 32% said they viewed a recovered school pupil as a risk to other students (Focus 1000, 2014).

Although challenges—including stigma—relating to the experience of survivors, their families and healthcare workers in Ebola case management facilities have been explored, less attention has been paid to the experiences and needs of those engaged as front-line responders in primary care facilities where many cases first present (Cooper 2015; Deng *et al.* 1978; Hewlett and Hewlett 2005; Hughes 2015; Khan *et al.* 1999; Kinsman 2012; Pathmanathan *et al.* 2014). These individuals are among the most vulnerable to infection and are at an exceptionally high risk for being (or having been) stigmatized, ostracized, attacked, contracting Ebola or forced to bear witness to tremendous human suffering (Hewlett and Hewlett 2005; Kinsman 2012). In Sierra Leone, preliminary estimates presented in February 2015 suggest that at least 65% of healthcare worker infections occurred among providers working in non-Ebola-specific care facilities (Bennet 2015). Ensuring the protection of workers in Ebola-specific facilities was prioritized in the beginning of the outbreak; however, this approach did not account for the fact that the non-specific clinical presentation of the disease meant that most Ebola patients seeking care first visited a non-Ebola healthcare centre. Health care workers in primary health care facilities and hospitals where therefore particularly vulnerable to contracting Ebola (Levy *et al.*, 2015).

Given a paucity of research on the psychosocial context of Ebola, and limited insights drawn from the experiences of providers – particularly those working in peripheral health facilities – this article examines how front-line providers in Sierra Leone experienced and assessed the changes in their professional, personal and social lives during the protracted Ebola outbreak (Hughes, 2015).

## Materials and Methods

### Study setting

The study took place in eight peripheral health units (PHU) across two districts (Bo and Kenema) in Sierra Leone during the height of the 2014–15 Ebola epidemic. PHUs were government-run and included Community Health Posts (CHP), Maternal and Child Health Posts (MCHP) and Community Health Centers (CHC). Qualified staff ranged from two to five clinical personnel at each facility. The total catchment population for PHUs included in the study is 35 738 and 16 240 in Bo and Kenema districts, respectively. During this period, upwards of 300 new cases were confirmed weekly (WHO 2014a).

### Study design

This analysis draws from 54 qualitative, semi-structured interviews with 35 providers including community health officers, nurses, maternal child health aides, community health workers and laboratory technicians. Communities were primarily Mende, and most lower tier health professionals identified as Mende. Higher tier health professionals were from a variety of ethnic backgrounds, but many were also Mende speakers. These research activities were conducted as a part of a larger mixed methods study on improving infection prevention and control in PHUs in the context of Ebola (2015). At the outset of the overall study, the research team intended to interview each provider twice to assess possible changes after a workshop to develop infection prevention and control improvement plans, but this was not always feasible as providers could be absent, ill or providing care on the day of the scheduled interview. Interviews typically lasted 1 hour. This participatory study was not designed to explore the psychosocial dimensions of Ebola. However, in response to questions about Ebola case management, impressions of Ebola, and knowledge or experiences implementing infection prevention measures, respondents discussed feeling emotionally, socially and physically distanced from colleagues, families, friends or the broader community. Following the iterative nature of qualitative research, this theme was more purposefully explored as interviews progressed.

### Data collection

Data were collected between 15 December 2014 and 30 January 2015. A team of eight data collectors with previous experience in research on behalf of NGOs or community development sectors was trained for 3 days, including 1 day of piloting and tool refinement. Four of the data collectors identified as Mende: three as Temne and one as Fullah. Free and informed consent was obtained from each respondent prior to the start of interviews. Interviews were audiorecorded, conducted and transcribed verbatim in Krio or Mende before being translated into English. Respondent characteristics are outlined in Table 1.

**Table 1.** Respondent characteristics

	Bo (*n* = 16)	Kenema (*n* = 19)
Male	5 (31)	10 (53)
Age		
<30	4 (25)	4 (21)
30–39	4 (25)	7 (37)
40–49	5 (31)	6 (32)
≥50	2 (13)	1 (5)
Missing	1 (6)	1 (5)
Job title		
Community health assistant	4 (25)	0
Community health officer	1 (6)	2 (11)
Community health worker	1 (6)	0
Maternal child health aide	5 (31)	4 (21)
State enrolled nurse	4 (25)	7 (37)
Laboratory technician	0	1 (5)
Other	1 (6)	4 (21)
Missing	0	1 (5)

### Data analysis

An initial phase of open, inductive coding on a selection of rich, diverse and representative transcripts was done by the lead author, as informed by principles of Grounded Theory (Charmaz 2006). This resulted in the creation of a codebook that was shared by the lead author and validated by a senior author (L.H.). The codes were then applied to remaining transcripts using Dedoose (Lieber and Weisner 2013). The lead author routinely provided analytic summaries to the study leads (L.H. and H.B.) and received feedback that helped to further refine the analysis and inform higher-level interpretations of the work.

### Ethical approval

The study received ethical approval from Institutional Review Boards of Durham University and the Sierra Leone Ethics and Scientific Review Committee.

## Results

We first present an overview of how providers described ‘living through Ebola’. We then present experiences at the PHU^1^, community, household and individual levels (Table 2).

**Table 2.** Provider experiences of social, emotional and physical distancing in the “Time of Ebola”

In health facilities
Changes in facility routines and practices
Facility quarantines; restriction of movement within facilities (including infection prevention screening)
Changes in provider-provider relations
No communal eating; loss of trust among providers
Changes in patient-provider relations
No touching, holding or hugging a grieving patient, maintaining distance from and among patients, isolating ill patients, “turning one’s shoulder” to patients, denying emergency care until a patient has been screened
In communities
Restrictions or bans against
Communal or public gatherings (for school, burials, meetings, soccer matches and construction projects)
Travel
Entering/exiting communities
Burning of houses or possessions of Ebola patients
At home
Restrictions or bans against
Sitting close to others
Handshaking or hugging
Checking in on neighbors or accepting visitors
Checking on sick family or friends
Children’s movements
Intimate relationships
As individuals
Providers report
Grief, loneliness, suffering and sadness
Feeling stigmatized by family and community
Fearing death

One theme that respondents consistently articulated was the manner in which Ebola undermined a sense of trust within and across health facilities, communities and families. Many providers described a need to be vigilant and ‘not trust’ others because of the fear of contracting Ebola, which ‘lives not only in your enemies but can get you through close relationships … with your friends, wife, children and family’. This sentiment applied to anyone with whom a provider came into contact, from family members to patients to colleagues. Providers described needing to constantly ‘observe’ those around them to watch for strange or infection-compromising behavior. One provider explained that she used to hold lactating mothers’ babies during weigh-ins but since the Ebola outbreak, ‘No one will take your child because we do not trust you. Providers do not even trust one another. We nurses do not get close to patients because there is no trust’. A lack of trust was especially prominent in encounters with strangers or visitors from another town, and to community members who had been away a period of time because, as one respondent put it, ‘We cannot allow you to be among us because we don’t know where you are coming from, if you have fallen sick and you come to us, we will never know’.

### Distancing within facilities

Two facilities included in this study underwent a 21-day quarantine due to early, compromised contact with a confirmed Ebola case. In those facilities, providers described being ‘deprived of our movement’ and feeling ostracized, scared and upset. Describing that period, one respondent said, ‘We did not even have access to even our own children. We were lying on the floor for 21 days’. The quarantine induced panic within the facilities and the broader community; one provider reported that ‘community members tried to burn this place down’. Although the quarantine had ended by the time of interviews, providers described a tense ‘waiting period’ post-quarantine as clients did not return to the facility for fear of contracting Ebola.

Although facilities included in the study were not under quarantine at the time of the study, providers described how ‘movement’ and a sense of ‘being free’ was restricted due to the erection of fencing and screening booths (also called ‘interrogation tables’). Providers understood the necessity of the screening process, but were profoundly upset by the fact that it prevented them from immediately treating urgent cases. Providers reported that many individuals suffering from an illness in the post-Ebola period delayed seeking care until their cases were severe because they viewed facilities as Ebola transmission points. This meant that more severe cases were presenting at facilities, including ‘a delivering woman’, ‘patients with bleeding wounds’, ‘shaking children’, ‘shivering brothers and sisters’, ‘dying people’ and individuals who were so ill that they had difficulty walking. As one provider said, ‘If we have not done triage, we are not going to take care of that person even if she is in labor… we have to abandon her on the street’. Along with these extreme situations, providers also described imposing necessary but ‘disliked’ measures to physically separate patients, such as telling them ‘not to cluster’ and to stay an arms-length distance away from other patients. They also had to stop women from co-nursing babies.

Providers described how they missed casual interactions with the community. ‘People used to be happy to come here, to meet us, to say thanks. The place was open … Now the hospital is fenced off’. Another provider said, ‘Nobody comes to … say hello anymore’. Still another presented this comparison: ‘Children used to come here and hug us. Now they run away from us… they call us the devil’. In one facility, providers described how they used to be ‘blessed’ with a heavy caseload including ‘people who came from far away, even though they had nearby facilities’. Given restrictions on travel, and bans against allowing strangers into a community or one’s home, providers said they could no longer ‘open our hands to you if you are not from this community’.

#### Strained relationships between providers

Relationships between providers were also described as strained. When asked to discuss life in the months since the outbreak began, one provider said she felt that ‘although we are in this together, there is a distance between us’. Another said she and her colleagues felt constant anxiety due to the concern that ‘you make one little mistake’ and you may infect yourself, your colleagues or your family. Providers said they no longer touched one another, or gathered to converse or eat; that they were instructed to observe and ‘watch over’ one another for infection-compromising behavior; and that previous feelings of trust and confidence had been replaced by the sentiment that ‘it is every man for him or herself’ and ‘an infection to one … can be an infection to all’. Some lower grade health workers, such as porters, reported that senior health workers monopolized the use of protective material such as gumboots, leaving them unprotected in their work. In an extreme case, a nurse described how a colleague survived Ebola, but upon returning home and learning that his wife and children had died, he ‘became paralyzed’ and ‘lost his mind’. The nurse said her only option was to call an ambulance to take her colleague back to the Ebola treatment unit as facility providers were ‘scared to get near him’ or touch him.

#### Strained patient–provider relationships

Among the most painful adjustments providers described since the onset of the Ebola outbreak related to patient–provider relationships. Providers described ‘preaching and living the don’t-touch practice’ and knowing that ‘too much sympathy will lead to Ebola infection’, but they nevertheless missed touching their patients, holding or hugging a grieving patient, assisting lactating mothers with their babies, sitting face-to-face or ‘being near’ patients, and rushing to assist individuals presenting with an emergency. Providers felt bad that infection prevention equipment ‘dehumanized’ them in the face of patients, and that ‘thermometer guns’ (used to take temperatures at screening booths) initially terrified many community members. One provider used the phrase ‘turning one’s shoulder’ to describe how she felt while interacting with patients.

I feel bad because I am a medical person, and this disease is preventing us from touching patients…. I am an MCH [maternal child health] Aide and I always carry out deliveries and immunization … I must touch my patients.

This stood in contrast to previous facility practices wherein providers ‘were open, welcoming’, ‘would check patients with our bare hands’, and would ‘suffer alongside’ patients. One provider felt she was no longer connected to her patients: ‘It used to be that patients would just come to the center without being screened, and we would allow that patient to enter. Whether the patient came with a contagious disease or not all of us will just suffer through it’.

In extreme (and rare) instances, respondents described how patients cursed, slapped and attacked providers because they heard rumors of providers ‘injecting Ebola’ and ‘selling bodies’ as a means for personal financial gain.

### Distancing within communities

Along with drastic changes in their professional lives, providers also described changes within their communities. One provider said, ‘nothing is working any more’ and another described how Ebola was ‘wiping away’ the progress Sierra Leone had made in the post-war period. Providers described how it could be traumatizing for a community to watch as an Ebola victim’s personal possessions were burned, leaving ‘nothing but walls’. Providers said protracted school closures were becoming problematic as children were left with little to occupy their days; one provider feared that his ‘bored’ daughter would ‘get into trouble’—possibly even become pregnant—if schools were not re-opened soon. Providers said they missed attending burials and visiting their sick families. Travel bans were described as necessary (‘because Ebola is everywhere in the country’). Nevertheless, bans prevented providers from visiting sick, dying or grieving family members, which was especially discomforting given that providers were often called upon to assist ailing relatives.

Three people have died in my hometown. I’ve not gone there to pay a visit. I have decided not to attend any burial because if I do, when I come back whether it's Ebola or not anything that happens to me even if it’s just a slight headache they will say I went to the village to attend a burial.

One provider described missing soccer and his favorite team, Manchester United: ‘Watching soccer (at the community television) was my hobby.… I am really unhappy because I no longer see my players when they are playing’. Along with community television cancellations, broader public meetings and celebrations were also cancelled as well as building projects (including a hospital expansion project and a latrine construction). One exception was religious services, which were permitted to continue.

### Distancing at home - within and across households

Similar restrictions on movement and interactions were also experienced in the context of interactions at home and with neighbors. Providers said they avoided hugging or handshaking, ‘even if it is your sister whom you have not seen for a long time’ or your ‘son who is visiting’. Providers described telling members of their household to ‘stop going to another person’s compound’, and they discussed how they tried in vain to restrict their children’s movement. One provider said she imposed limits on the distance her child was allowed to be from their home, but he routinely disobeyed her orders. Another said she forced her children to sit inside. Still another described how her child no longer had friends because neighbors ‘quietly stigmatized’ the children of health providers.

In instances where providers could not prevent visitors from coming to their homes, they described setting up washing stations and requiring that guests remove their shoes. More often though they discouraged such home visits or confined visitors to designated areas of their property.

Providers also described the impact of Ebola on intimate relationships. One provider said that since a man she had been dating was away for several weeks, she knew she could not ‘allow him (near me) until I know his status’. Other providers said they had learned to ‘avoid sexual intercourse. There were so many love relationships that have stopped because of Ebola’.

### Personal perceptions and experiences of distancing

The changes described in their professional lives, communities and households affected how providers felt about themselves and their social surroundings. Providers described ‘suffering’, ‘loneliness’, being ‘isolated’ and feeling ‘full of sadness’ since the onset of Ebola. Many said they were in the midst of grieving the deaths of wives, husbands, sisters, children, colleagues and friends. One provider said she was ‘bleeding in my heart for all of my colleagues’ who had died. Another stated, ‘We have a saying that there is nothing that can break a family, but this sickness will cut an entire family. It destroys everything’.

Providers said they yearned for ‘the way things were before Ebola’. They felt that they were ‘not trusted’, ‘not loved’, and ‘not respected by the community’, that they had ‘fewer friends’, and that people wanted to ‘keep a distance’ from them. Providers said they heard neighbors ‘whispering things’ about them and were viewed with suspicion. One provider used the phrase ‘killing my spirit’ to describe how the community’s perception of her made her feel. She said she had tried to address this problem by saying:

If you are scared of me, it makes me feel bad. And what if I feel bad and get angry and decide not to go to the center again? What if all the health workers sit down and refrain from treating Ebola patients? Who will do that job? People have come from other countries to help us fight Ebola. If we sit in our own country and say we will not take part in that fight how will the disease go?

Several providers noted their families stopped coming near them or talking to them due to fear. Many of their families urged them to stop working at health facilities. Several providers said they did, at some point, leave their posts, which was a demoralizing and guilt-inducing decision. One provider felt lonely at the end of the workday because she went home and had to ‘sit alone and isolate myself’ from her family. Another provider said he felt that he ‘owns the Ebola’ because his family told him, ‘You, the health workers, currently we are afraid of you because you possess the disease’. Another provider said she felt distrusted by her family. She shared the following account from a conversation with her mother:

As for my mother I do not go to her, nor does she come to me. When she needs something, I send it to her… Even the last time we were talking, I was just making a joke and I said that I want to pay her a visit and she kept saying, ‘If you come right now, we will quarantine you for 21 days before we allow you to mix with us’ and then I told her, ‘It’s just a joke. I don’t even have plans to go’.

Finally, providers described fearing their own mortality. ‘I have begun to imagine my own death, how sad my family would be without me’, said one. They described losing their appetite, having trouble sleeping, and ‘living in fear’.

## Discussion

This article documents the experiences of front-line providers working in primary healthcare settings during the recent Ebola outbreak in Sierra Leone. Such providers have received relatively little attention compared with those working in Ebola treatment units or Ebola patients and their families despite their critical role in the epidemic response. Amid the Ebola outbreak, the Sierra Leonean Ministry of Health and Sanitation recognized the risk to peripheral health providers and prioritized improvements to infection prevention measures across the country's 1200 PHUs (Levy *et al.*, 2015). The timely deployment of equipment coupled with the continued operation of > 95% of these facilities at the height of the epidemic reflects an impressive commitment across multiple levels of the health system (Levy *et al.* 2015). Nevertheless, this study has highlighted providers' concurrent need for support and guidance on how to cope with the stress, horror, danger, grief and human misery that enveloped them in their role as *de facto* first responders in the Ebola crisis (WHO, World Vision, UNICEF 2014). Like providers in Ebola treatment units, our respondents internalized the suffering they experienced as witnesses to sudden, horrifying deaths from Ebola (Padickakudi *et al.* 2015). The ways in which providers know that wearing protective equipment is necessary, but makes them feel inhuman or uncaring has also been discussed in relation to this and other outbreaks (Borchert *et al.* 2007; Cooper 2015). Despite facing these significant stressors, providers commendably continued to work out of a sense of dedication to their jobs, their patients and their communities.

Ethnic cleavages between healthcare providers and communities experiencing epidemics have been identified in other settings as exacerbating effective epidemic management (Bolten 2014). In our study neither ethnic difference nor the gender of health workers emerged as a significant points of tension. We found that the primary cause of unease and distrust was being a health worker. This experience was shared across all tiers of health workers, and by para-health professionals (such as porters). Providers in our study described feeling stigmatized by people to whom they had previously been very close, including friends, their own family members, fellow health professionals and the broader community. Previous research on Ebola outbreaks has emphasized that many of the measures introduced by affected communities such as increasing distance and avoiding contact can aid disease control (Hewlett and Amola 2003; Hewlett and Hewlett 2007). However, the effect of these measures upon health workers was distressing. Health workers were accustomed to working in stressful and traumatic situations, without proper resources, sometimes without pay, and dealing with a range of dangerous infectious diseases. However, many had worked and lived among the communities whom they served for many years and spoke movingly of radical transformation in interpersonal relations that they experienced during the Ebola outbreak. Despite this, they remained committed to their efforts as health professionals. Similar feelings of stigma have been reported by providers in previous Ebola outbreaks in Uganda, Republic of Congo and Democratic Republic of Congo (Hewlett and Hewlett 2005; Kinsman 2012). In Sierra Leone, our findings suggest that initial conspiracy beliefs that assigned discrediting attributes to providers were not sustained later in the epidemic. Rather, the protracted stigma described by providers appeared to be linked to fears of Ebola transmission and nosocomial infection. However, as the epidemic evolves, more pointed research into the nature of stigma and the potential emergence of courtesy stigma, or stigma by association, may merit further investigation across types of health facilities and types of providers (Phillips and Benoit 2013).

Psychological literature has highlighted that individuals affected by Ebola may feel compounded grief as their ability to cope is constrained; individuals such as those in this study cannot engage in rituals that facilitate grieving (such as burials) or practices that demonstrate bonding (such as physical touch) (WHO, World Vision, UNICEF 2014). In an effort to mitigate psychiatric morbidities and maladaptive coping mechanisms, and to prevent the spread of Ebola, we urge research on ways to provide psychosocial support (through messaging, media and health system supports) to providers across various strata of the healthcare system—not only to those working in Ebola treatment units (Hughes 2015). This should include attention to indigenous strategies developed by health workers and members of the communities whom they serve, which have been introduced to mitigate the effects of these experiences and to improve relations with service users. The WHO's provisional psychological guidance emphasizes the importance of psychological first aid, or ‘human, supportive and practical help’, to those suffering in an Ebola crisis (WHO, World Vision, UNICEF 2014). The guidance states that individuals need to feel safe, listened to, comforted and capable of accessing support to help themselves and their communities during and immediately after an Ebola outbreak (WHO, World Vision, UNICEF 2014).

Such recommendations need to be articulated through the specific needs and experiences of affected communities. As a starting point for formative research, we urge engaging with providers and community members to identify and pilot interventions that are deemed feasible and culturally acceptable in order to mitigate distress. Such interventions could draw from existing mental health literature that emphasizes the provision of counseling for providers (particularly those who are placed under quarantine); mandatory rest periods for health workers (coupled with supportive supervision) and the designation of a mental health professional who can work with providers in the months after an acute or critical incident to assist in healing (Hughes 2015; IASC 2010). In 2015, the WHO issued guidelines to assist non-specialist, first-line providers in their efforts to address mental health needs amid humanitarian emergencies (WHO 2015b). Although these guidelines emphasize measures that providers can undertake to meet patient needs, the process of creating an atmosphere that promotes mental health and provides tools for managing stress, grief and depression may help to empower providers (WHO 2015b). We are encouraged by Sierra Leone’s launch of a mental health policy and strategic plan, and hope that this will bolster an expansion of efforts to address mental healthcare needs (WHO 2012). According to a 2012 WHO report, Sierra Leone has severe shortages in human resources for mental health; the ratio of psychiatrists, psychiatric nurses, neurologists and social workers is 0.02, 0.04, 0.02 and 0.06, respectively, per 1000 people (Alemu *et al.* 2012). The report stated that there are no neurosurgeons or psychologists in the country (Alemu *et al.* 2012). Related to Ebola prevention specifically, further efforts to make personal protective equipment more humanizing, such as by adding pictures of providers’ faces to the front of their suits (Aizenman 2015) or having providers don equipment in the community, rather than arriving fully dressed (Raabe *et al.* 2010), may also enhance patient–provider bonding (Aizenman 2015; Hughes 2015).

Although we applaud recent calls to improve the overall health system (Kieny *et al.* 2014) and to address critical shortfalls in human resources for health, our research demonstrates that the psychosocial needs of existing health workers merit immediate attention. Although emotional and social support—as well as acute psychological care—are often neglected amid disease outbreaks, the importance of mental health was poignantly documented in an account of an Ebola treatment unit's suspension of services in Monrovia, Liberia (one of two functioning units at the time) due to a provider's psychotic episode (Cooper 2015).

## Limitations

In this article, we present qualitative data on the experiences of front-line Sierra Leonean primary healthcare providers during the recent Ebola outbreak. Such providers play a critical role in outbreak responses yet have received relatively little attention in the academic literature. We conducted interviews during the height of the 2014–15 outbreak, allowing for a timely analysis. Nonetheless, our findings must be considered in light of limitations. In terms of transferability, we interviewed providers from eight health facilities in two predominantly Mende districts in the south and east of the country, and our results cannot necessarily be generalized to providers in other settings or other outbreaks. The themes discussed in this article emerged from a broader study on infection control. As we did not design the study to specifically consider the psychosocial effects of the epidemic on health workers, there are limitations in the scope of our material. Areas that merit further consideration include how experiences of this epidemic compared with other forms of stress experienced by health workers, and how characteristics of the outbreak such as caseload and epidemic curve affect provider experience. Similarly, although ethnicity, gender, age and professional position did not emerge as factors that compelled community unease towards providers, we did not probe specifically for these variables.

## Conclusion

Ebola has torn at the fabric of Sierra Leonean society. It has highlighted the vulnerability of a fragile health system and revealed underlying tensions in this post-conflict setting. Literature related to Ebola generally, and to the current epidemic in West Africa, emphasizes the epidemiology of the disease, including clinical manifestations. We argue that efforts to address this and other stigmatizing diseases must explicitly incorporate an examination of the social landscape of the disease and its mental health consequences. These efforts must extend their focus to include front-line healthcare workers.

In this and future epidemics, responses must consider that front-line healthcare providers are among the earliest to be affected by an outbreak and they may be subjected to intense and protracted fear and stigmatization by the very communities in which they live and treat patients. Community engagement and sensitization should not only address knowledge dissemination related to transmission but also incorporate modules that are attentive to the psychosocial needs of providers and their communities. This would involve explicitly promoting non-discrimination and non-stigmatization towards health providers, victims and victims' families (De Roo *et al.* 1998) and providing special support to providers. In much of west Africa, access to mental health and psychosocial service is limited whereas infectious disease epidemics remain relatively common, which highlights a need to address issues such as those presented in this study.

## Notes

1 In Sierra Leone, it is common practice to use the word “hospital” when referring to any health facility. We have retained the term hospital when used by respondents, but highlight that this wording is in reference to peripheral health units, which provide first-line, primary health care.
